# Interplay of Gastrointestinal Parasites, Micronutrient Deficiencies, and Anemia in Children from the Bolivian Highlands

**DOI:** 10.3390/microorganisms14020511

**Published:** 2026-02-22

**Authors:** Washington R. Cuna, Roberto Passera, Celeste Rodriguez

**Affiliations:** 1 Unidad de Inmunología Parasitaria, Facultad de Medicina, Universidad Mayor de San Andrés, La Paz 10077, Bolivia; washingtoncuna@gmail.com; 2 Department of Medical Sciences, University of Torino, 10126 Torino, Italy; passera.roberto@gmail.com; 3 Academia Nacional de Ciencias de Bolivia, La Paz, Bolivia

**Keywords:** children, anemia, intestinal parasites, micronutrient deficiency, zinc, vitamin A, vitamin D, Bolivia

## Abstract

Children living in resource-limited regions with inadequate environmental sanitation, such as the Bolivian highlands, are affected by parasitic infections that may compromise nutritional status. **Objective:** This study aimed to assess the prevalence of intestinal parasitic infections and their associations with nutritional status, micronutrient deficiencies, and anemia in school-aged children from La Paz, Bolivia. **Methods:** A cross-sectional study was conducted among 212 schoolchildren aged 5–13 years in the municipality of La Paz, in highland areas characterized by high poverty levels. Parasitological examination, anthropometric measurements, and biochemical assessment of micronutrients (vitamins A and D, zinc, iron) were performed to evaluate children’s health status. **Results:** Mild malnutrition was more prevalent than moderate-to-severe forms. Micronutrient analysis revealed substantial deficiencies in vitamin A (39%), zinc (25%), and vitamin D (18%). Zinc deficiency was significantly more common in children aged 11–13 years compared to younger age groups (*p* = 0.034). Intestinal protozoan infections showed significant associations with micronutrient deficiencies. *Giardia lamblia* infection was associated with both vitamin A (30.9%, *p* = 0.042) and vitamin D (78.9%, *p* = 0.001) deficiencies. *Blastocystis* spp. infection was similarly linked to higher prevalence of vitamin A (35.8%, *p* = 0.025) and vitamin D (69.7%, *p* = 0.004) deficiencies. *Entamoeba coli* infection was significantly associated with vitamin D deficiency (*p* = 0.021), while *Iodamoeba bütschlii* infection showed a significant association with zinc status (*p* = 0.027), with notably lower zinc deficiency prevalence in infected children (7.7%) compared to non-infected children. Among helminth infections, *Ascaris lumbricoides* was significantly associated with vitamin D deficiency (37%, *p* = 0.018). Moderate-to-severe anemia was highly prevalent, affecting over half of the children regardless of sex. Wasting (BAZ) was significantly associated with age (*p* = 0.030), with moderate-to-severe cases most prevalent in children aged 5–7 years and absent in older groups, while mild wasting increased with age. In univariate logistic regression analysis, zinc deficiency emerged as a significant risk factor for anemia (OR = 2.51, 95% CI: 1.19–5.29, *p* = 0.016). No significant associations were observed between anemia and sex, age group, vitamin A or D status, or anthropometric indicators including underweight, stunting, or wasting. **Conclusions:** These findings highlight the substantial burden of micronutrient deficiencies, parasitic infections, and anemia among children in this impoverished region, underscoring the urgent need for targeted public health interventions addressing nutritional supplementation, parasite control, and improved sanitation.

## 1. Introduction

Intestinal parasitic infections remain a major public health concern in low- and middle-income countries, particularly affecting children living in resource-limited and high-altitude regions such as the Bolivian highlands [1,2]. These infections persist where environmental sanitation, hygiene, and access to clean water are inadequate, contributing substantially to the global burden of childhood undernutrition [3,4]. Beyond their immediate health effects, intestinal parasites can impair intestinal function, alter the gut microbiota, and induce chronic inflammation, thereby compromising nutrient absorption and utilization [5]. The disruption of nutrient absorption caused by parasitic infections is especially detrimental during early childhood, a critical period for growth and cognitive development. Helminths such as *Hymenolepis nana* and *Ascaris lumbricoides*, commonly reported in Andean populations, interfere with the absorption of fats and fat-soluble vitamins (A and D), leading to diarrhea, weight loss, and anemia [2,6,7]. Similarly, protozoan infections including *Giardia lamblia* and *Blastocystis hominis* provoke intestinal inflammation and mucosal damage that reduce the uptake of essential minerals such as zinc and iron [8,9,10]. These nutrient deficiencies weaken immune function, delay physical growth, and heighten susceptibility to further infections, perpetuating a vicious cycle of malnutrition and disease. Micronutrient deficiencies, the inadequate intake of essential vitamins and minerals, represent one of the most prevalent forms of malnutrition globally, disproportionately affecting children in developing countries due to limited access to nutritious food [11,12,13,14]. Often termed “hidden hunger”, micronutrient deficiencies lack the visibly signs of other forms of undernutrition, making them difficult to detect until serious health consequences emerge [15]. These deficiencies may go undetected for long periods, with the severity and extent of the deficiency determining its sequalae. Therefore, evidence-based strategies are crucial to prevent micronutrient deficiencies, particularly during childhood, a critical period of rapid growth and development. Nonetheless, the associated consequences can have serious and lasting effects on physical and cognitive development, resulting in poor health, stunted growth, cognitive impairments, and reduced earning potential later in life [16,17].

Anemia represents another major consequence of both parasitic infections and micronutrient deficiencies in vulnerable pediatric populations. While iron deficiency is the primary cause of anemia globally, deficiencies in other micronutrients including vitamins A and D, and zinc, can also impair erythropoiesis and hemoglobin synthesis [18,19]. National data on the micronutrient status of Bolivian children remain scarce. However, previous reports documented a high anemia prevalence among children in impoverished settings, characterized by limited dietary diversity and widespread chronic undernutrition [1,20]. Given the likely burden of micronutrient deficiencies in this context, this study focused on assessing deficiencies in vitamins A and D, and zinc. Vitamin A is essential for vision, cellular differentiation, and both humoral and cell-mediated immunity; deficiency can lead to visual impairment and compromised immune function [21]. Vitamin D plays critical roles in bone development and immune modulation, including regulation of macrophage and dendritic cell activity, cytokine production, and mucosal defense mechanism [22,23,24]. Similarly, zinc is crucial for multiple organ systems and physiological processes, especially during periods of rapid growth and development. Deficiency during these critical windows can result in cognitive impairments, learning difficulties, and an increased risk of neurological disorders [25,26,27].

Characterizing the complex interplay between anemia, micronutrient deficiencies and parasitic infections is therefore essential for understanding child health in impoverished Bolivian communities. This comprehensive approach enables the development of targeted, multifaceted interventions to improve nutritional status and health outcomes in at-risk children.

## 2. Materials and Methods

### 2.1. Study Area, Population, and Assessment

Cross-sectional quantitative data were collected from schoolchildren across twelve school units in the municipality of La Paz, La Paz department, Bolivia, located at altitudes ranging from 3810 to 4050 m above sea level. These highland communities are characterized by high poverty levels, limited access to clean water, and inadequate sanitation infrastructure, including limited access to toilets and wastewater treatment systems. These conditions collectively contribute to poor child nutritional and health outcomes, particularly elevated rates of micronutrient deficiencies and anemia among children.

A total of 212 children aged 5 to 13 years participated in the study and provided blood samples for the assessment of vitamin A, vitamin D, zinc, and hemoglobin levels.

### 2.2. Measurements of Vitamin A, Vitamin D, and Zinc

Peripheral serum samples were analyzed for vitamin A levels using a competitive enzyme immunoassay (MyBiosource, Inc., San Diego, CA, USA). Vitamin A concentrations below 0.70 μmol/L were classified as deficient, levels between 0.70 and 1.05 μmol/L indicated marginal deficiency, and concentrations above 1.05 μmol/L reflected adequate vitamin A status [28,29].

25(OH)-vitamin D concentrations in serum were determined using a commercial enzyme-linked immunosorbent assay (ELISA) kit, following the manufacturer’s instructions (Immundiagnostik AG, Bensheim, Germany). Published cut-offs were used to define serum vitamin D levels as adequate (>75 nmol/L), insufficient (50–74 nmol/L), or deficient (<50 nmol/L) [30].

Morning fasting serum zinc levels were measured using a colorimetric assay kit (MyBiosource, Inc., San Diego, CA, USA). Zinc deficiency in children was defined as a serum zinc level of <70 µg/dL [31].

### 2.3. Hemoglobin Determination

Venous whole blood was collected, and hemoglobin (Hb) levels were measured using an HemoCue hb201+ analyzer (Angelholm, Sweden). Anemia was diagnosed according to age-specific hemoglobin cut-offs adjusted for altitude (3930 m) by adding 2.5 g/dL to sea-level cut-offs (11.5 g/dL for ages 5–11 years; 12.0 g/dL for ages 12–13 years), resulting in adjusted cut-offs of 14.0 and 14.5 g/dL, respectively. Severity was classified as mild (11.2–13.9 and 11.6–14.4 g/dL), moderate (8.4–11.1 and 8.7–11.5 g/dL), and severe (<8.4 and <8.7 g/dL) for each age group [32].

### 2.4. Anthropometric Measurements

Trained local nurses took anthropometric measurements (i.e., height and weight) to assess the children’s nutritional characteristics. Weight-for-Age Z-score (WAZ), height-for-age (HAZ), and body mass index-for-age (BAZ) Z-scores were calculated using WHO AnthroPlus software (version 1.0.4) for children aged 5–19 years, based on the 2007 World Health Organization (WHO) growth reference standards [33]. Children with a WAZ ≤ −2 score standard deviations (SD) were defined as underweight, those with HAZ ≤ −2 SD as stunted, and those with BAZ ≤ −2 SD as wasted. Mild underweight, mild stunting, or mild wasting were defined as <−1 Z score. The study was approved by the ethics committee of the Universidad Mayor de San Andrés in La Paz, Bolivia.

### 2.5. Determination of Parasitic Infections

Parasites were detected through microscopic examination of stool samples; initial screening used direct wet mounts (saline and Lugol’s iodine), with two slides examined per sample at 100× magnification. Positive samples underwent formalin–ethyl acetate concentration (Ritchie). Wright stain was used solely for samples with clinical suspicion of amebic dysentery to rapidly identify hematophagous trophozoites and differentiate *E. histolytica* from non-pathogenic amoebae in fresh preparations. *Entamoeba histolytica* infection was confirmed through the observation of hematophagous trophozoites containing ingested red blood cells. We assessed only the presence of helminth infections, not their intensity. Individuals were classified as positive for gastrointestinal parasites when diagnostic stages (eggs, larvae, cysts, or trophozoites) were observed during microscopic examination. All material was analyzed independently by two researchers.

### 2.6. Statistical Methods

Participant characteristics were analyzed using Fisher’s exact test for categorical variables and the Mann–Whitney test for continuous ones; the continuous variables have been described as median [interquartile range (IQR)], while the categorical ones as absolute/relative frequencies. A series of univariate binary logistic regression models was estimated to investigate the potential role of several determinants (age/sex/vitamin A deficit/vitamin D deficit/zinc deficit/WAZ, HAZ and BAZ alterations) on the main outcome (occurrence of a moderate/severe anemia). All reported *p*-values have been obtained by the two-sided exact method, at the conventional 5% significance level. Data have been analyzed as of 10 July 2025 by R 4.5.1 (R Foundation for Statistical Computing, Vienna-A, http://www.R-project.org, Accessed as of 10 July 2025).

## 3. Results

### 3.1. Study Population

Table 1 presents the demographic and nutritional characteristics of the study population. The sex distribution was relatively balanced, with 53.1% females and 46.9% males. Children aged 8–10 years represented the largest age group (41.6%). Due to missing values, anthropometric data were available for 180 to 212 children depending on the indicator. Mild forms of malnutrition were more prevalent than moderate/severe forms, with 7.7% of children underweight, 27.3% stunted, and 10.4% wasted. These findings point to a substantial burden of mild nutritional deficiencies in this high-altitude population.

### 3.2. Micronutrient Deficiencies

Vitamin A deficiency was the most prevalent micronutrient deficiency, observed in 39.0% of the children, followed by zinc (25.0%) and vitamin D (18.0%) (Table 2). Rates of deficiency were similar between females and males across all micronutrients. By age group, zinc deficiency increased significantly with age, from 15.5% in 5–7-year-olds to 36.6% in 11–13-year-olds (*p* = 0.034). Although the prevalence of vitamin A and D deficiencies also varied by age, particularly marginal vitamin A deficiency and vitamin D insufficiency, these differences were not statistically significant.

### 3.3. Associations Between Parasitic Infections and Micronutrient Status

Table 3 presents the relationships between individual parasitic infections and micronutrient deficiencies. Among helminth infections, only *Ascaris lumbricoides* showed a significant association with altered vitamin D status (*p* = 0.018), with infected children displaying a higher proportion of vitamin D deficiency (37.0%).

Several protozoan infections demonstrated significant associations with micronutrient deficiencies. *Giardia lamblia* infection was significantly associated with both vitamin A deficiency (*p* = 0.042) and vitamin D status (*p* = 0.001), with 78.9% of infected children presenting vitamin D deficiency. *Blastocystis hominis* infection showed significant associations with both vitamin A (*p* = 0.025) and vitamin D status (*p* = 0.004), with 69.7% of infected children exhibiting vitamin D deficiency. *Entamoeba coli* infection was also significantly associated with vitamin D status (*p* = 0.021). Additionally, *Iodamoeba butschlii* infection showed a significant association with zinc status (*p* = 0.027), with infected children demonstrating a notably lower prevalence of zinc deficiency (7.7%) compared to non-infected children.

### 3.4. Anemia and Anthropometric Measurements

The prevalence of anemia and the anthropometric indicators weight-for-age (WAZ), height-for-age (HAZ), and BMI-for-age (BAZ) by sex and age group among children is shown in Table 4. Moderate to severe anemia affected more than half of both girls (54.9%) and boys (57.1%). Regarding anthropometric indicators, no significant differences were found between sexes. Overall wasting (BAZ) showed a significant age-related trend (*p* = 0.030), with moderate to severe wasting most prevalent among children aged 5–7 years and absent in the oldest age groups, while the prevalence of mild wasting increased with age. Younger children were more likely to be underweight; however, this association was marginally significant (*p* = 0.054), suggesting a possible decline in prevalence with increasing age.

Table 5 presents the results of univariate binary logistic regression models evaluating potential determinants of moderate/severe anemia, which was observed in 117 participants (56.0%) of the total study cohort. Zinc deficiency was the only factor significantly associated with moderate/severe anemia, with affected children having more than twice the odds of anemia compared to those with adequate zinc levels (OR = 2.51; 95% CI: 1.19–5.29; *p* = 0.016). No significant associations were found for sex, age group, or deficiencies in vitamin A or vitamin D. Although vitamin A deficiency was associated with higher odds of anemia (OR = 1.63; *p* = 0.112), the association did not reach statistical significance. Similarly, none of the anthropometric indices (WAZ, HAZ, BAZ) were significant predictors of anemia, either in its mild or moderate/severe forms.

## 4. Discussion

This study sought to investigate the complex interplay between micronutrient status, growth outcomes, and anemia in the context of gastrointestinal parasitic infections in resource-limited settings where micronutrient deficiencies are common. We found substantial prevalence rates of vitamin A (39.0%), zinc (25.0%), and vitamin D (18.0%) deficiencies, alongside a high burden of stunting (33%) and anemia exceeding 50% among children across all age groups. Remarkably, zinc deficiency increased significantly with age, rising from 15.5% in the youngest group (5–7 years) to 36.7% in the oldest group (11–13 years). Strong associations were observed between specific parasitic infections, particularly *G. lamblia*, *Blastocystis* spp., and *A. lumbricoides,* and deficiencies in fat-soluble vitamins.

### 4.1. Contextualization of Findings Within the Broader Literature

To appropriately interpret these findings, it is essential to situate them within the existing literature and understand the biological mechanisms driving micronutrient deficiencies in this unique high-altitude setting. Our observed prevalence of vitamin A deficiency (39%) is considerably higher than rates reported from other Latin American countries, including Mexico (25%) [34] and Peru (11.7%) [35], but remarkably similar to prevalence estimates from sub-Saharan Africa (48%) and South Asia (44%) [36]. This similarity to resource-limited settings in other continents, despite geographical distance, suggests shared underlying determinants that transcend regional boundaries. In contrast, our zinc deficiency prevalence (25%) is notably lower than the 57.1–67.5% reported in comparable populations in sub-Saharan Africa [37] and also lower than rates documented in Colombia (56.3% in indigenous and 42.1% in nonindigenous populations) and Guatemala (41.2% in indigenous and 29.8% in nonindigenous children) [38]. The vitamin D deficiency rate of 18% observed in our study is particularly striking given Bolivia’s high solar radiation due to its proximity to the equator and high altitude. However, this finding is consistent with recent reports from other high-altitude populations, where cultural practices such as extensive clothing coverage for sun protection, darker skin pigmentation limiting cutaneous synthesis, and complex high-altitude UV dynamics may substantially reduce vitamin D production despite abundant sunlight exposure [39]. These quantitative differences across geographical settings underscore the critical importance of context-specific nutritional assessment rather than extrapolating prevalence estimates from geographically or culturally distant populations, even when environmental conditions such as altitude or poverty levels appear comparable.

### 4.2. Underlying Mechanisms of Micronutrient Deficiencies

These quantitative differences across geographical settings are not merely statistical variations but reflect distinct underlying biological and environmental processes. The high prevalence of micronutrient deficiencies in our study population likely reflects multiple interacting mechanisms. Dietary factors play a central role: the traditional highland Bolivian diet is predominantly plant-based with limited animal-source foods, resulting in low bioavailability of zinc and preformed vitamin A due to high phytate content in staple grains and limited heme iron sources [40]. Altitude-related factors at >3600 m may influence both dietary absorption and metabolism; chronic hypoxia induces adaptations in iron metabolism and erythropoiesis that may alter micronutrient requirements [41]. Parasite-mediated mechanisms contribute substantially: *Giardia* infection damages intestinal brush border membranes and reduces fat absorption, directly impairing uptake of lipid-soluble vitamins A and D [42,43]; our finding of strong associations between *Giardia* and both vitamin A and D deficiencies supports this mechanism. Similarly, *Ascaris* infection can cause villous atrophy and increased intestinal permeability, potentially explaining the observed association with vitamin D deficiency [44]. The inflammation-nutrient interaction represents an additional pathway: chronic parasitic infections trigger systemic inflammation, which downregulates retinol-binding protein synthesis and redistributes zinc to acute-phase proteins, effectively reducing bioavailable pools of these micronutrients even when dietary intake may be adequate [45]. The convergence of these dietary, environmental, and infection-related mechanisms in our population creates a particularly high-risk context for multiple concurrent micronutrient deficiencies.

### 4.3. Micronutrient Deficiencies and Growth Patterns

Against this backdrop of converging risk factors, the specific nutritional and growth patterns observed in our study take on particular significance. The high burden of stunting (33%) in this population points to chronic nutritional deprivation, with the significant increase in zinc deficiency with age providing a key mechanistic insight for this growth failure. This finding is consistent with research in similar settings that identifies zinc as a critical factor limiting linear growth. However, zinc deficiency represents just one facet of the high prevalence of mild nutritional deficiencies observed in this study, which highlights a significant yet frequently overlooked public health issue in children. Although these deficits may not present with overt clinical signs, suboptimal levels of key micronutrients such as vitamin A, zinc, and vitamin D can compromise immune function, hinder cognitive development, and impair physical growth, with effects that may persist into adolescence and adulthood [46,47,48,49,50]. In our setting, the substantial burden detected aligns with recent global estimates indicating that hundreds of millions of children, particularly in low- and middle-income countries, experience one or more micronutrient deficiencies [47]. The insidious nature of these deficiencies, often referred to as “hidden hunger”, poses challenges for early detection and timely intervention, as children may appear clinically well despite having biochemical or mild anthropometric deficits [15,16,17]. The observed growth patterns are primarily driven by inadequate access to diverse and nutritious food during this critical period of rapid growth and are consistent with reports from other low- and middle-income settings, where school-aged children frequently present with multiple micronutrient insufficiencies despite the absence of overt malnutrition [11]. The age-related increase in zinc deficiency observed here demonstrates the need for age-targeted nutrition strategies, particularly for older children who may be more vulnerable due to higher nutrient demands and dietary inadequacies [25]. Notably, among children with vitamin A deficiency, 43.6% were also deficient in vitamin D, underscoring the clustering of micronutrient deficiencies. Given the critical roles of vitamin A and zinc in immune competence, and vitamin D in both immune function and bone health, such deficiencies can increase susceptibility to infections, impair physical development, and reduce cognitive performance [12,48,49,50,51].

The significant age-related trend of decreasing moderate-to-severe wasting, alongside increasing mild wasting, likely reflects partial recovery from acute undernutrition in early childhood, coupled with persistent low-grade nutritional deficits and chronic conditions such as environmental enteropathy [52]. Younger children in our study appear more susceptible to severe wasting, due to higher infection risk, inadequate dietary intake during weaning, and the acute effects of pathogen exposure in unsanitary environments [1]. As they grow older, improved resilience and care may reduce severe cases, yet ongoing exposure to poor water, sanitation, and hygiene conditions can sustain subclinical intestinal inflammation, impair nutrient absorption, and limit full catch-up growth, resulting in persistent mild wasting [5].

### 4.4. Parasite-Specific Associations with Micronutrient Deficiencies

The mechanisms outlined above provide important context for interpreting the distinct associations observed between specific protozoan infections and nutritional deficiencies in our study. A particularly telling finding was the specific co-association of *G. lamblia* infection with deficiencies of the fat-soluble vitamins A and D. The fact that infected children exhibited drastically significant higher rates of both vitamin A (30.9%, *p* = 0.042) and vitamin D (78.9%, *p* = 0.001) deficiency points to a shared pathophysiology. This pattern is highly consistent with a state of fat malabsorption, a known pathophysiological effect of *G. lamblia* that damages the intestinal mucosa [53]. The consequent impairment of lipid-soluble vitamin absorption compromises both immune functions, mediated by vitamins A and D, and bone health, which is dependent on vitamin D [48,50].

The often-overlooked protozoan *B. hominis* was also strongly associated with nutritional deficiencies, particularly in vitamin D (69.7%, *p* = 0.004) and vitamin A (35.8%, *p* = 0.025). This suggests that *B. hominis*, frequently dismissed as a commensal, may have a substantive impact on host nutritional status. The specific impairment of fat-soluble vitamin absorption mirrors our findings for *G. lamblia*, indicating a potential shared mechanism. This malabsorption is likely mediated by damage to the intestinal wall, as *B. hominis* infection has been observed to increase intestinal permeability and is correlated with significantly lower concentrations of vitamin A [10,53,54]. Other protozoa infections by *E. coli* and *I. bütschlii* were significantly associated with vitamin D (*p* = 0.021) and zinc (*p* = 0.027) deficiencies, respectively. The observed association between *E. coli* colonization and vitamin D deficiency is initially surprising, given its well-established status as a non-pathogenic and clinically insignificant commensal. This finding challenges the simplistic dichotomy of pathogenic versus non-pathogenic parasites. The seemingly paradoxical association may be explained by a model in which *E. coli* is not a direct etiological agent of the deficiency, but instead a marker of underlying pathology or a factor in subclinical gut dysregulation. A high *E. coli* load is a clear marker of fecal–oral contamination, often associated with poor sanitation and hygiene. These same conditions are risk factors for other subclinical gastrointestinal infections and environmental enteric dysfunction, a condition of chronic gut inflammation and malabsorption, which could directly impair the absorption of fat-soluble vitamins like vitamin D [55]. The observed link between *I. bütschlii* and zinc deficiency may similarly be indirect. Like *E. coli*, this non-pathogenic parasite is an unlikely direct etiological agent. Instead, its presence may serve as a sensitive marker of broader gut ecosystem disturbances, including altered microbial diversity or mucosal function, that ultimately compromise zinc absorption.

The helminths *H. nana* and *A. lumbricoides* showed fewer and weaker associations. The finding that children infected with *A. lumbricoides* had significantly lower vitamin D deficiency compared to non-infected children is paradoxical and difficult to interpret, particularly given the widespread vitamin D deficiency (>50%) observed across all age groups in our study population. There is no established biological mechanism by which *A. lumbricoides* infection would improve vitamin D status. On the contrary, ascariasis has been causally linked to vitamin A deficiency and malabsorption [56,57], and intestinal helminths are generally associated with nutrient malabsorption rather than improved nutritional status. This unexpected association may reflect unmeasured confounding factors, residual confounding by socioeconomic or behavioral variables not fully captured in our analysis, or reverse causality whereby better vitamin D status influences susceptibility to or clearance of *A. lumbricoides* infection. Future longitudinal studies with detailed assessment of potential confounders, infection intensity, and temporal sequences would be necessary to clarify whether this association represents a genuine biological relationship or spurious association.

### 4.5. Anemia and Zinc Deficiency

This study found a high prevalence of anemia exceeding 50% among children across all age groups, with no significant variation by sex or age. This represents a serious public health concern given the adverse consequences for physical and cognitive development and increased morbidity risk in school-aged children [58,59,60]. The concurrent finding of low prevalence of moderate-to-severe malnutrition suggests that anemia in this population is likely attributable to micronutrient deficiencies rather than general undernutrition. Limited access to iron-rich foods such as meat, combined with diets dominated by carbohydrates, vegetables, and fruits containing absorption inhibitors (phytates and polyphenols), further compromises iron and zinc bioavailability in children [61]. Regression analysis revealed that zinc had the strongest association with hemoglobin levels, suggesting that zinc deficiency contributed to the anemia observed in children [62]. Zinc deficiency has been linked to anemia in other populations of children [63], with proposed mechanisms including its role as an intermediary between selenium and hemoglobin synthesis [64]. However, the effect of zinc on anemia may depend on interactions with other coexisting micronutrient deficiencies. This multifactorial nutritional scenario is particularly concerning in highland children, as the combined effects of anemia and zinc deficiency threaten physical growth, immune function, cognitive development, and overall health. Further research is needed to elucidate the synergistic impact of zinc deficiency alongside other micronutrient deficits in the etiology of childhood anemia in resource-limited settings.

### 4.6. Strengths, Limitations, and Future Directions

This study has several limitations. We did not collect dietary intake data (iron, zinc, vitamin A, fats), socioeconomic indicators (household income, parental education), inflammation markers (CRP, AGP), or deworming history. The absence of inflammation markers may have led to misclassification of micronutrient deficiency status, as acute phase response can distort biomarker levels independent of true nutritional status. We assessed only the presence of parasitic infections, not their intensity, which may have limited our ability to detect dose–response relationships. Additionally, our cross-sectional design precludes causal inference.

### 4.7. Conclusions and Public Health Implications

Our findings contribute novel evidence to the limited body of literature examining the intersection of intestinal parasitic infections and micronutrient deficiencies in school-aged children and adolescents from endemic settings. While global datasets have extensively documented protein-energy malnutrition and its association with increased infection risk, far less attention has been paid to specific micronutrient deficiencies in parasitized populations, particularly for this age group in Latin America. The present study addresses this gap by providing comprehensive biochemical data on zinc, vitamin A, vitamin D, and iron status alongside detailed parasitological assessment in Bolivian children and adolescents, a population for which such integrated data are virtually absent from the published literature. Importantly, our biochemical approach reveals nutritional vulnerabilities that would not be detected through anthropometric assessment alone, as micronutrient deficiencies can exist even in children with normal weight-for-age or height-for-age indices. The high prevalence of anemia and micronutrient deficiencies observed among school-aged children in the Bolivian highlands, associated with dietary inadequacies, environmental factors, and gastrointestinal parasitic infections, underscores the urgent need for integrated interventions combining deworming, micronutrient supplementation, and dietary diversification tailored to this population. Critically, our findings demonstrate that the determinants of childhood malnutrition are context-specific rather than universal; the patterns observed in the Bolivian highlands likely differ substantially from those documented in sub-Saharan Africa or Southeast Asia. This context-specificity highlights the essential role of localized research in informing effective, evidence-based public health policies and interventions in diverse geographical and cultural settings, extending the value of our work well beyond a regional description to contribute meaningful insights for global nutrition and parasitology efforts in endemic areas.

## Figures and Tables

**Table 1:** Demographic and anthropometric characteristics of children from the Bolivian highlands.

Characteristics	N/Total (%)
Demographic	
Sex (%)	
Female	111/209 (53.1)
Male	98/209 (46.9)
Age (%)	
5–7	52/209 (24.9)
8–10	87/209 (41.6)
11–13	70/209 (33.5)
Nutritional status	
Underweight	
mild	14/180 (7.7)
moderate/severe	8/180 (4.4)
Stunting	
mild	58/212 (27.3)
moderate/severe	12/212 (5.7)
Wasting	
mild	22/212 (10.4)
moderate/severe	8/212 (3.8)

Underweight, stunting, and wasting classified according to WHO child growth standards using Z-scores: mild (Z-score −1 to −2 SD), moderate/severe (Z-score < −2 SD, severe < −3 SD). SD, standard deviations.

**Table 2:** Prevalence of micronutrient deficiencies by sex and age group among children in the Bolivian highlands.

Vitamin A Status, n (%)					
Characteristic	n	Adequate	Marginal Deficiency	Deficiency	*p* *
Overall	200			78 (39.0)	
Sex					
Female	106	54 (50.9)	12 (11.3)	40 (37.7)	
Male	94	50 (53.2)	6 (6.4)	38 (40.4)	0.509
Age group					
5–7 years	50	29 (58.0)	4 (8.0)	17 (34.0)	
8–10 years	84	44 (52.4)	10 (11.9)	30 (35.7)	
11–13 years	66	31 (47.0)	4 (6.1)	31 (35.7)	0.464
**Zinc Status, n (%)**					
**Characteristic**	**n**	**Adequate**		**Deficient**	*p* *
Overall	176			44 (25.0)	
Sex					
Female	100	76 (76.0)		24 (24.0)	
Male	76	56 (73.7)		20 (26.3)	0.729
Age group					
5–7 years	45	38 (84.4)		7 (15.5)	
8–10 years	71	56 (78.9)		15 (21.1)	
11–13 years	60	38 (63.3)		22 (36.7)	**0.034**
**Vitamin D Status, n (%)**					
**Characteristic**	**n**	**Adequate**	**Insufficient**	**Deficient**	*p* *
Overall	206			37 (18.0)	
Sex					
Female	110	28 (25.4)	17 (15.4)	65 (59.1)	
Male	96	21 (21.9)	20 (20.8)	55 (57.3)	0.558
Age group					
5–7 years	51	15 (29.4)	5 (9.8)	31 (60.8)	
8–10 years	86	23 (26.7)	14 (16.3)	49 (57.0)	
11–13 years	69	11 (15.9)	18 (26.1)	40 (58.0)	0.116

*p* *-values indicate significance of differences between gender and age groups based on Fisher’s exact test. Significant associations (*p* * < 0.05) highlighted in bold.

**Table 3:** Associations between parasitic infections and micronutrient status in children from the Bolivian highlands.

Vitamin A Status, n (%)					
Parasite	n/N	Adequate	Marginal Deficiency	Deficiency	*p* *
*Helminths*					
*Hymenolepis nana*	46/192	21 (45.7)	4 (8.7)	21 (45.7)	0.723
*Ascaris lumbricoides*	27/200	18 (66.7)	0 (0.0)	9 (33.3)	0.111
*Protozoa*					
*Entamoeba coli*	105/197	51 (48.6)	8 (7.6)	46 (43.8)	0.377
*Endolimax nana*	20/191	11 (55.0)	1 (5.0)	8 (40.0)	0.885
*Giardia lamblia*	68/196	44 (63.2)	4 (5.9)	21 (30.9)	**0.042**
*Blastocystis hominis*	95/198	57 (60.0)	4 (4.2)	34 (35.8)	**0.025**
*Iodamoeba butschlii*	35/195	19 (54.3)	1 (2.9)	15 (42.9)	0.407
*Chilomastix mesnili*	45/196	29 (64.4)	1 (2.2)	15 (33.3)	0.059
*Entamoeba histolytica/dispar*	22/192	15 (68.2)	2 (9.1)	5 (22.7)	0.155
**Zinc Status, n (%)**					
**Parasite**	**n/N**	**Adequate**		**Deficient**	*p* *
*Helminths*					
*Hymenolepis nana*	45/166	34 (75.6)		11 (24.4)	0.844
*Ascaris lumbricoides*	19/174	16 (84.2)		3 (15.8)	0.409
*Protozoa*					
*Entamoeba coli*	93/171	71 (76.3)		22 (23.7)	0.599
*Endolimax nana*	18/165	15 (83.3)		3 (16.7)	0.408
*Giardia lamblia*	67/170	52 (77.6)		15 (22.4)	0.475
*Blastocystis hominis*	92/172	71 (77.2)		21 (22.8)	0.387
*Iodamoeba butschlii*	26/169	24 (92.3)		2 (7.7)	**0.027**
*Chilomastix mesnili*	41/170	29 (70.7)		12 (29.3)	0.683
*Entamoeba histolytica/dispar*	19/166	16 (84.2)		3 (15.8)	0.407
**Vitamin D Status, n (%)**					
**Parasite**	**n/N**	**Adequate**	**Insufficient**	**Deficient**	*p* *
*Helminths*					
*Hymenolepis nana*	49/198	15 (30.6)	6 (12.2)	28 (57.1)	0.321
*Ascaris lumbricoides*	27/206	7 (25.9)	10 (37.0)	10 (37.0)	**0.018**
*Protozoa*					
*Entamoeba coli*	108/203	26 (24.1)	27 (25.0)	55 (50.9)	**0.021**
*Endolimax nana*	20/197	3 (15.0)	7 (35.0)	10 (50.0)	0.138
*Giardia lamblia*	71/202	8 (11.3)	7 (9.9)	56 (78.9)	**0.001**
*Blastocystis hominis*	99/204	16 (16.2)	14 (14.1)	69 (69.7)	**0.004**
*Iodamoeba butschlii*	35/201	6 (17.1)	9 (25.7)	20 (57.1)	0.351
*Chilomastix mesnili*	46/202	7 (15.2)	8 (17.4)	31 (67.4)	0.219
*Entamoeba histolytica/dispar*	23/198	3 (13.0)	7 (30.4)	13 (56.6)	0.198

n (%), stands for number and percentage of infected children within each micronutrient category. n/N: infected children/total tested for the parasite. *p* *-values from Fisher’s exact test, comparing status distribution between infected and non-infected children. Significant associations (*p* * < 0.05) in bold.

**Table 4:** Prevalence of anemia and anthropometric measurements by sex and age group in children of the Bolivian highlands.

	Female	Male	*p* *	5–7 Years Old	8–10 Years Old	11–13 Years Old	*p* *
Anemia							
mild	19.8%	18.3%		9.6%	24.1%	20.0%	
moderate/severe **	54.9%	57.1%	0.946	63.5%	55.2%	51.4%	0.222
Anthropometrics							
WAZ							
mild	6.4%	9.3%		12.9%	7.9%	0.0%	
moderate/severe	3.2%	5.8%	0.536	7.4%	4.5%	0.0%	0.054
HAZ							
mild	23.7%	31.6%		26.4%	22.5%	34.3%	
moderate/severe	4.4%	7.1%	0.238	5.7%	4.5%	7.1%	0.431
BAZ							
mild	6.1%	15.3%		7.5%	10.1%	12.8%	
moderate/severe	3.5%	4.1%	0.085	11.3%	2.2%	0.0%	**0.030**

* *p*-value reflects the significance of differences in prevalence by sex and age group, using Fisher’s exact test. ** Severely anemic: Hb < 8.7 g/dL. Anthropometrics: according to WHO Z-scores, mild < −1 SD, moderate < −2 SD, severe < −3 SD Significant associations (*p* * < 0.05) in bold.

**Table 5:** Binary logistic regression univariate models for evaluation of determinants of anemia in children of Bolivian highlands.

Determinants	OR (95% CI)	*p* *
Sex (male vs. female)	1.09 (0.63–1.89)	0.751
Age (yrs)		0.410
(8–10) vs. (5–7)	0.71 (0.35–1.43)	0.338
(11–13) vs. (5–7)	0.61 (0.29–1.27)	0.186
Micronutrients		
Vitamin A deficiency		0.251
(marginal vs. adequate)	0.96 (0.35–2.62)	0.940
(deficient vs. adequate)	1.63 (0.89–2.96)	0.112
Zinc deficiency		
(deficient vs. adequate)	**2.51 (1.19–5.29)**	**0.016**
Vitamin D deficiency		0.410
(insufficient vs. adequate)	0.64 (0.27–1.51)	0.304
(deficient vs. adequate)	1.05 (0.54–2.06)	0.887
Anthropometrics		
WAZ-defined underweight		0.501
(mild vs. normal)	0.92 (0.31–2.79)	0.887
(moderate/severe vs. normal)	0.42 (0.10–1.80)	0.240
HAZ-defined stunting		0.449
(mild vs. normal)	0.73 (0.39–1.34)	0.306
(moderate/severe vs. normal)	1.45 (0.42–5.05)	0.559
BAZ-defined wasting		0.864
(mild vs. normal)	0.83 (0.34–2.06)	0.688
(moderate/severe vs. normal)	0.76 (0.18–3.11)	0.697

Significant associations (*p* * < 0.05) in bold.

## Data Availability

The original contributions and data presented in this study are included in the article.
